# Endothelial cell alignment as a result of anisotropic strain and flow induced shear stress combinations

**DOI:** 10.1038/srep29510

**Published:** 2016-07-12

**Authors:** Ravi Sinha, Séverine Le Gac, Nico Verdonschot, Albert van den Berg, Bart Koopman, Jeroen Rouwkema

**Affiliations:** 1 Department of Biomechanical Engineering, MIRA Institute for Biomedical Technology and Technical Medicine, University of Twente, Enschede, The Netherlands; 2 Applied Microfluidics for BioEngineering Research group, MIRA Institute for Biomedical Technology and Technical Medicine, MESA+ Institute for Nanotechnology, University of Twente, Enschede, The Netherlands; 3 Radboud university medical center, Radboud Institute for Health Sciences, Orthopaedic Research Lab, P.O. Box 9101, 6500 HB, Nijmegen, The Netherlands; 4 BIOS, Lab on a chip group, MIRA Institute for Biomedical Technology and Technical Medicine, MESA+ Institute for Nanotechnology, University of Twente, Enschede, The Netherlands

## Abstract

Endothelial cells (ECs) are continuously exposed *in vivo* to cyclic strain and shear stress from pulsatile blood flow. When these stimuli are applied *in vitro*, ECs adopt an appearance resembling their *in vivo* state, most apparent in their alignment (perpendicular to uniaxial strain and along the flow). Uniaxial strain and flow perpendicular to the strain, used in most *in vitro* studies, only represent the *in vivo* conditions in straight parts of vessels. The conditions present over large fractions of the vasculature can be better represented by anisotropic biaxial strains at various orientations to flow. To emulate these biological complexities *in vitro*, we have developed a medium-throughput device to screen for the effects on cells of variously oriented anisotropic biaxial strains and flow combinations. Upon the application of only strains for 24 h, ECs (HUVECs) aligned perpendicular to the maximum principal strain and the alignment was stronger for a higher maximum:minimum principal strain ratio. A 0.55 Pa shear stress, when applied alone or with strain for 24 h, caused cells to align along the flow. Studying EC response to such combined physiological mechanical stimuli was not possible with existing platforms and to our best knowledge, has not been reported before.

Endothelial cells reside in a mechanically active environment and are known to be highly responsive to mechanical stimuli. They are continuously exposed to cyclic circumferential strain and shear stress from the pulsatile blood flow. *In vitro* studies have shown that when exposed to these mechanical stimuli, endothelial cells distinctly change in morphology as compared to static cultures. They align perpendicular to the strain direction when uniaxial strain^1^ is applied and along the flow direction in response to flow induced shear stresses^2^, which resembles the morphology found *in vivo* in the presence of these stimuli^3^. The mechanical stimuli also affect the cellular function. Both strains^4^ and shear stresses^5^ are known to regulate the permeability of endothelial cell monolayers, thus affecting one of their major functions of serving as a barrier between blood and surrounding tissue. Besides existing blood vessels, strains^6^ and shear stresses^7^ also play an important role in the formation of new vessels (angiogenesis). More importantly, disturbed mechanical stimuli can lead to endothelial dysfunction. Disturbed flows near bends or branching in blood vessels leading to low and/or oscillating shear stresses are widely recognized as a cause leading to atherosclerosis^8^. Similarly, strains have been linked to atherosclerosis^9^.

*In vitro* platforms to apply well-defined mechanical stimuli to cells have played a critical role in understanding how strains and shear stresses affect endothelial and other cells. Early research has been performed using macro scale platforms such as the commercially available Flexcell and several other custom built devices^10^. However these devices are fast being replaced by micro^11^,^12^ and meso^13^,^14^,^15^ scale devices that offer several advantages such as better control on the physical environment of cells, lower cell and reagent requirements, *in situ* imaging capabilities, high throughput potential and flexibility in design and fabrication permitting increasingly complex systems^16^. For endothelial cells, devices applying uniaxial strains in a flat substrate have been the most common as they mimic the circumferential strains found in blood vessels^17^. However, this simplification ignores the axial strains in blood vessels, which are usually small compared to the circumferential strains for straight parts of the vasculature in physiological conditions. However clinical observations and biomechanical studies have found that in disease states such as hypertension^18^, atherosclerosis^19^ or aneurysms^20^, the vascular wall changes in shape and mechanical properties, which in turn can lead to changes in the strains. A flat substrate undergoing anisotropic biaxial strains is a closer model to such a situation. For complex geometries, such as vessel branching regions, the anisotropic strains can better represent even the physiological conditions^17^. In many cases where strain anisotropies are present, the flow might not necessarily be perpendicular to the maximum strain direction and hence the strain and the shear stress do not direct the cells to align in the same direction. Hence, a combined anisotropic biaxial strain and shear stress producing system, which can also test for various orientations between the two stimuli, is essential to better address the biological complexity. The knowledge of the cell response to varying degrees of strain anisotropy, in combination with flow induced shear stress oriented at various angles to the strain, is relevant in understanding physiological or pathological cell behavior *in vivo,* or in predicting cell fate in a strain- and perfusion-stimulated cell seeded tissue engineering scaffold.

In this study, we have developed a medium throughput device to screen for the effects on cells of various anisotropic strains, each oriented at various angles to flow applied in combination with the strains. The device was developed using methods previously employed by us to design a separate device which could screen for the effects of various equibiaxial strain and various shear stress combinations^15^. The current device is similar in dimensions and the number of test units to the previous device but very distinct in the mechanical stimulation conditions that each device can test for (various anisotropic strains and flow at various orientations versus various equibiaxial strains and various shear stresses). The developed system has been used to study the effect of combined anisotropic surface strains and fluid flow shear stresses on human umbilical vein endothelial cells (HUVECs). Previously, combinations of anisotropic (uniaxial) strains and shear stresses have been only applied in situations where they complement each other in their alignment response, i.e. flow is applied perpendicular to the uniaxial/circumferential strain direction^21^,^22^,^23^,^24^, resembling flow and strain in straight parts of vasculature *in vivo*. To our knowledge, this is the first study investigating the alignment response of HUVECs to strains and shear stresses in complementary as well as non-complementary combinations.

## Results

### A device offering uniform anisotropic strains with varying degrees of anisotropy has been developed

**Figure 1:**
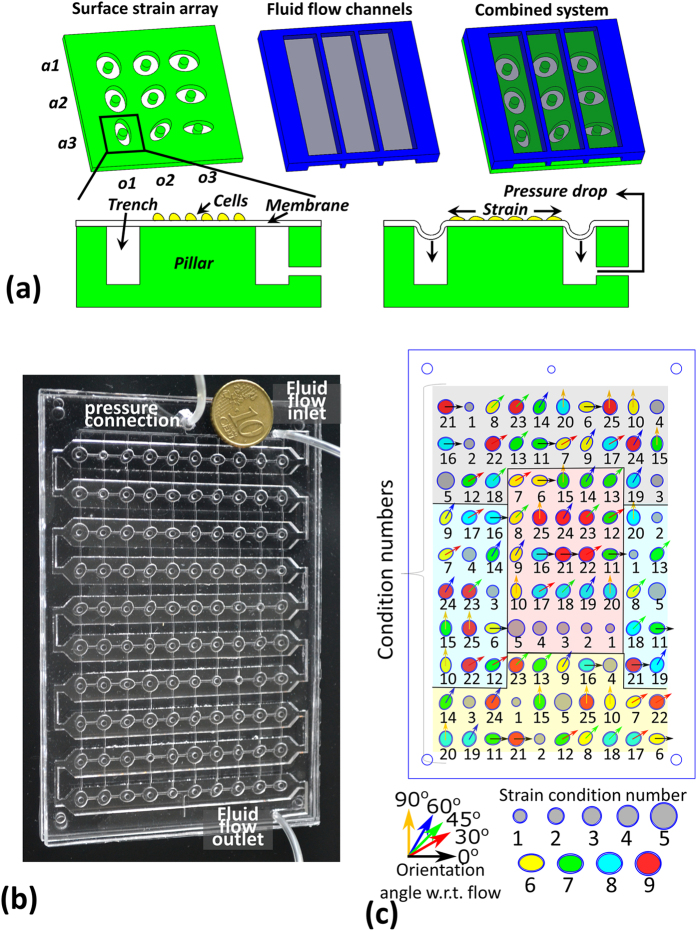
A schematic representation of the reported device (**a**) illustrates how strains are generated by stretching a membrane over a pillar and how these strains are combined with shear stresses using an overlaid flow channel. The schematic also shows how varying degrees of anisotropic strains (a1, a2, a3) are generated by using various aspect ratios of the strain actuating trenches, and that by using various orientations (o1, o2, o3) of the trench with respect to the flow channel, various orientations of the anisotropic strains with respect to the shear stresses can be tested. The developed prototype (**b**) and the layout of the various trench shapes (**c**) demonstrate how the various conditions are distributed randomly on the device to avoid location specific effects, while keeping a replicate of each condition in four regions that can be expected to differ. These four regions that the device is divided into are depicted in different colors – gray (near inlet), yellow (near outlet), blue (near bends) and red (mid-channel).

The developed device can screen for four degrees of anisotropy (maximum:minimum principal strain ratio) and five orientations of each anisotropic strain with respect to flow (total 20 conditions). The device can also screen for five equibiaxial strain magnitudes. Each of the 25 strain conditions is present in four replicates, giving a total of 100 test units. For ease of identification in the rest of the paper, the various conditions are assigned condition numbers, as listed in Table 1. The device consists of an array of 100 strain producing areas overlaid with a continuous winding flow channel with a single inlet and a single outlet. Strains are produced by pulling a thin elastic membrane over a circular pillar into an ellipse (anisotropic biaxial strain) or a circular (equibiaxial strain) trench using a pressure drop, as illustrated schematically in Fig. 1(a). The device has the base dimensions of standard well plates, permitting imaging automation using commercial bio-imagers such as the BD Pathway.

Computational modeling was used to design the device. Models of the strain production demonstrated that regions of uniform anisotropic surface strains can be produced by stretching a thin elastic membrane into an elliptical trench over a circular pillar using a pressure drop. At a given pressure drop, the degree of anisotropy could be varied by altering the ellipse major axis to minor axis ratio. The maximum and the minimum principal surface strains in the region over the pillar were directed along the ellipse trench major and minor axis directions respectively. In the central 50% surface area over the pillar, both the maximum and the minimum principal strain were largely uniform and hence this region was selected as the region of interest (ROI).

**Figure 2:**
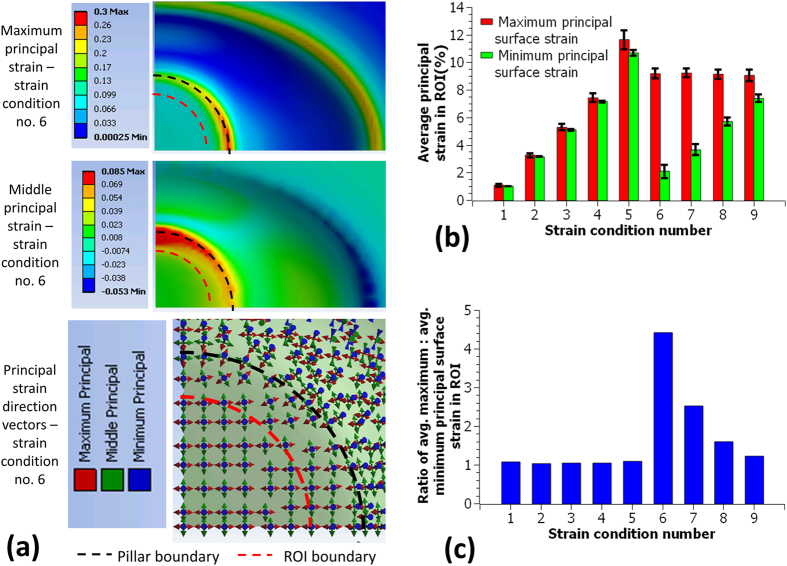
Finite element modeling of strain units showed that stretching a membrane over a circular pillar into an ellipse trench can generate uniform maximum and middle principal strains in the central 50% area over the pillar (ROI) (**a**). In the ROI the maximum and the middle principal strains were along the membrane surface and corresponded to the maximum and the minimum principal surface strains respectively. They were aligned along the trench major and minor axis respectively (**a**). The maximum and the minimum principal surface strains were averaged for the ROIs (**b**, bar plots displaying average ± standard deviations) and their ratios were calculated from the averages (**c**).

Figure 2(a) shows the ANSYS modeling results for the highest degree of anisotropy tested. Since the model was three dimensional, three principal strains (maximum, middle and minimum) were returned by ANSYS, where the minimum principal strain is perpendicular to the membrane plane and therefore not sensed by cells attached on the membrane surface. The maximum and the middle principal strain distributions in the membrane over the pillar and the trench regions, and the principal strain direction vectors over the pillar are plotted for an applied pressure drop of 20 kPa. In the ROI, the maximum and the middle principal strains from the 3D model lie along the membrane surface and correspond to the maximum and the minimum surface strains in the membrane respectively. The latter terminology will be used in the rest of the paper.

Based on these modeling results, geometries were selected to apply anisotropic strains with maximum:minimum principal strain ratios of 1.2, 1.6, 2.5 and 4.4 for a 20 kPa pressure drop; each with a maximum strain of ~9%. For the geometries selected for the equibiaxial strains, the model predicted strains were ~1, 3, 5, 7 and 11%. The modeling predicted principal strains and the principal strain ratios for all tested conditions are shown in Fig. 2(b,c) respectively.

**Figure 3:**
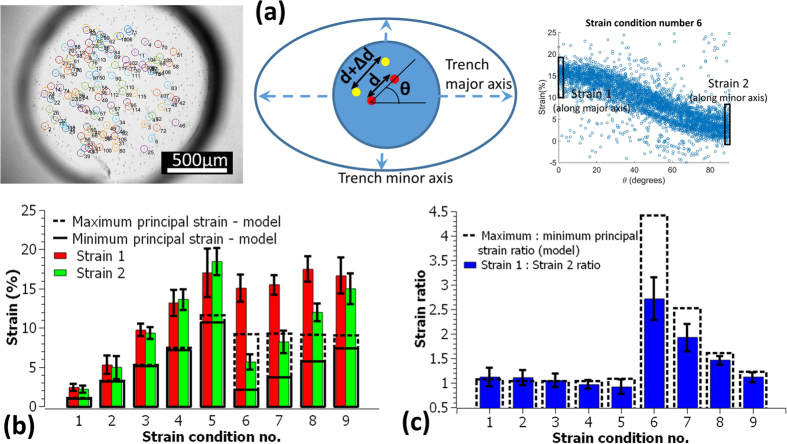
Empirical strain measurements were performed by tracking 6 μm beads embedded in the membrane. The beads were identified and mapped between strained and unstrained images of the membrane (**a**). Strains were calculated for all pairs of identified beads (Δd/d) and plotted against the angle that the bead pair made with the trench major axis (θ) (**a**). The strains for bead pairs oriented within 2.5° angles along the trench major and minor axis were averaged (strain 1 and strain 2 respectively) and used as approximations for the maximum and the minimum principal strains in the membrane. For the equibiaxial strain units, strain 1 and strain 2 were the averages along and perpendicular to the image horizontal direction respectively. The principal strains thus calculated (strain 1 and strain 2) were then averaged for each strain condition (**b**) (average ± standard deviations, n = 20 for the anisotropic strains and n = 4 for the equibiaxial strains). The principal strain ratios were also calculated and averaged for each strain condition (**c**) (average ± standard deviations, n = 20 for the anisotropic strains and n = 4 for the equibiaxial strains).

Strains that were empirically measured in the developed prototype by tracking beads embedded in the membrane, displayed the same trend as the model data but the strain values were higher than those predicted by the models. Similarly, the anisotropy ratios were affected as well. For a 20 kPa pressure drop, the empirically determined maximum principal strain for the anisotropic units was ~15% (Fig. 3(b)) and the average maximum:minimum principal strain ratios for the various types of units were ~1.1, 1.5, 1.9 and 2.7 (Fig. 3(c)). The equibiaxial strains were ~2, 5, 9, 13 and 17% (Fig. 3(b)) with maximum:minimum principal strain ratios between ~0.9 and ~1.1 (Fig. 3(c)).

Multiple factors can account for the mismatch between the models and the actual device, such as the use of inaccurate values for the friction coefficient between the pillar and the membrane, the membrane thickness, or the elastic modulus of polydimethylsiloxane (PDMS). Varying these factors in one of the models suggested that the elastic modulus value used is most likely causing the differences. Changing the elastic modulus in the model to a lower value of 1 MPa, which is on the lower side of the reported range in literature^25^, results in strain values close to the empirical results (Supplementary Figure 1).

### Flow induced shear stress in the device and its variation due to the strain actuating membrane dip were analyzed using computational fluid dynamics (CFD) modeling

**Figure 4:**
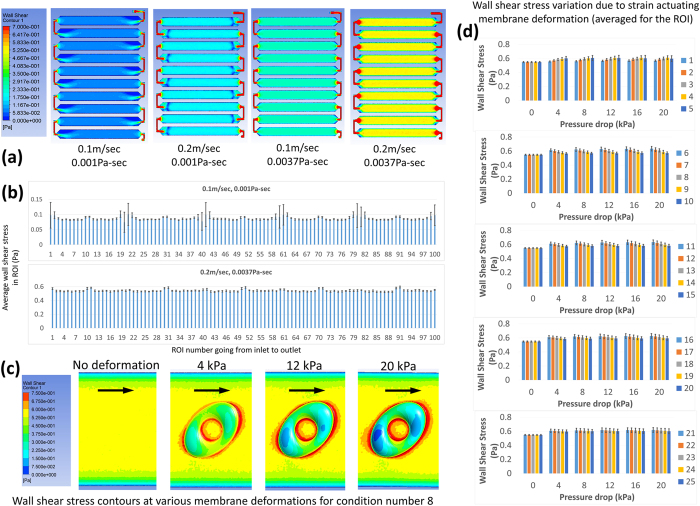
CFD models of the entire channel network were used to get the overall distribution of shear stress in the channel (**a**). Disturbances in the shear stress were observed in the regions near the flow channel bends when media alone was used. At a higher flow rate of 0.2 m/sec (corresponding to 40 ml/min), reversing flows were found near bends with media alone. However, when 5% (wt/wt) dextran added media was used, the disturbances near the bends were reduced and flow reversal near bends was avoided at the higher flow rate. The average shear stress in all ROIs was calculated from these models (**b**, average ± standard deviation). Local deformed geometries were used to assess shear stress variations due to the membrane dips during the strain application for an applied flow rate of 40 ml/min and a 0.0037 Pa.s viscosity (**c**, arrows indicate the flow direction). The shear stress was averaged for the ROIs for all deformed geometries (**d**, average ± standard deviation).

CFD results demonstrated that when cell culture media (viscosity 0.001 Pa.s) is flown through the flow channel, ROIs in the middle of the channel experience uniform shear stresses. However, those near the channel bends are exposed to disturbed shear stresses. The disturbances near the bends become small compared to the applied shear stress if the viscosity of the fluid is increased. These results are similar to our previous findings for a slightly different channel geometry^15^. To limit fluid flow variations and increase fluid flow shear stresses, media with 5% (wt/wt) dextran (viscosity ~0.0037 Pa.s) was used for the cell experiments. Figure 4(a) shows the shear stress contours for media alone and with added dextran, both for two inlet velocities −0.1 m/s and 0.2 m/s (corresponding to 20 ml/min and 40 ml/min volumetric flow rates). The 40 ml/min flow rate was used in experiments with the high viscosity media to keep the inflow higher than the total device volume change produced by the strain actuating membrane dips. For low viscosity media, however, the 20 ml/min flow rate was preferred in order to avoid reversing flows near bends, at the cost of flow reversal for ROIs near the outlet due to inflow not being able to fill up the increased device volume during strain actuation. Figure 4(b) shows the average shear stress in all ROIs for the two flow conditions tested in the experiments. The average shear stress values in the ROIs are 0.080 Pa for the media without dextran at 20 ml/min flow and 0.55 Pa for the media with 5% dextran at 40 ml/min flow.

Shear stress variations in the ROIs due to the membrane deformations were estimated using various deformed membrane geometries. The membrane deformations used were obtained from the strain models. Such an approach was chosen based on our previous observation that flow modeling with fixed geometries for the deformed membrane in a similar system^15^ predicted shear stress variations comparable to those predicted by more computationally expensive fluid structure interaction models. Figure 4(c) shows the shear stress contours for sample geometries and Fig. 4(d) shows the average shear stresses in the ROIs of the various test units for membrane deformations corresponding to five different pressure drops. Over half a strain cycle (i.e. 0 to 20 kPa pressure drop/0 to maximum strain) the average shear stress in the ROIs was found to increase with increasing membrane deformation (except for the 2.5 mm radius circular trench, where the shear stress in the ROI plateaued after an initial increase). For circular trenches, the increase in shear stress was higher for a bigger trench and for ellipse trenches, the shear stress increase was higher for the orientation with the bigger membrane deformation along the flow path before reaching the ROI (i.e. the increase was highest for the trench major axis aligned along the flow and lowest for the trench major axis aligned perpendicular to the flow). The biggest variation was observed for the condition number 6 where the average shear stress changed by 15.7% over a strain cycle.

### A ~0.55 Pa shear stress dominates surface strain in alignment response of HUVECs

**Figure 5:**
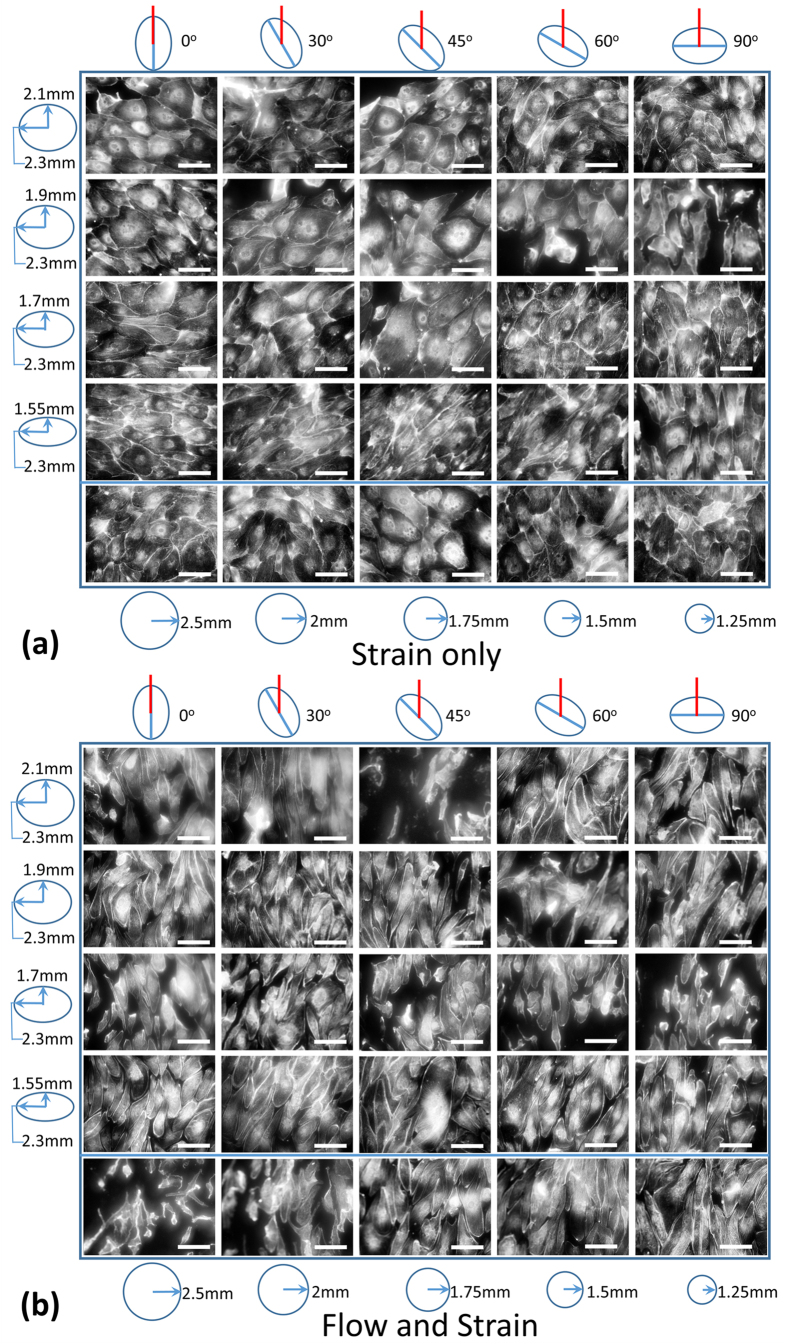
Fluorescent images of HUVECs stained with Alexa 488 phalloidin were taken using a long working distance 20× objective with BD Pathway for multiple (an)isotropic strain regimes at multiple angles to the fluid flow direction. The cells are shown after a 24 hour strain only (**a**) or a 24 hour flow and strain (**b**) stimulation under media containing 5% dextran (high viscosity). Scale bar −100 μm. In each image set, the conditions are arranged by the degree of anisotropy in the rows and by the orientation of the trench major axis in the columns. Ellipses of various aspect ratios drawn on the left depict the various degrees of anisotropy in the respective rows and the circles of various sizes drawn at the bottom depict the various equibiaxial strains in the last row. The ellipses drawn on the top of each image set represent the trench orientations while the red lines represent the flow direction or just the orientation of the flow channel (in the case of strain only stimulation).

**Figure 6:**
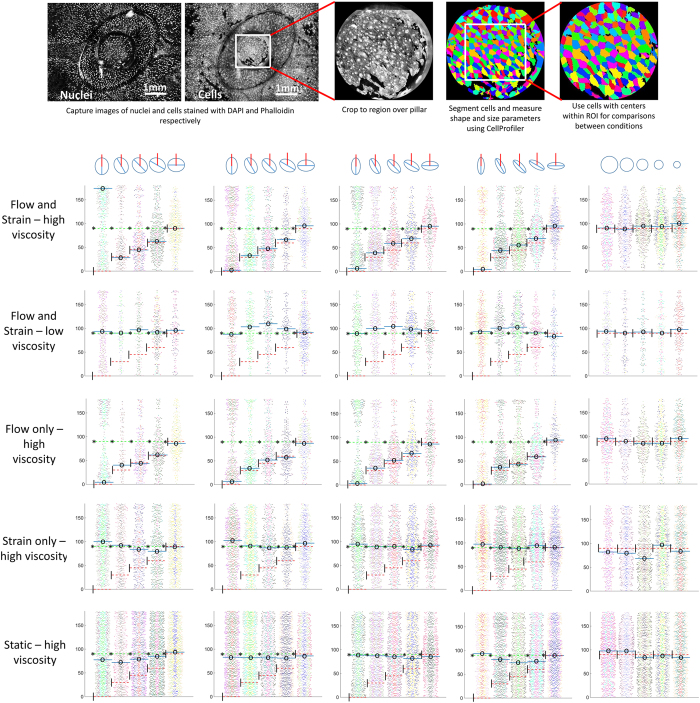
Images were also taken using a 4× objective with BD Pathway. 3 × 3 montages were captured for cells and nuclei stained with Alexa 488 Phalloidin and DAPI respectively. The montages captured complete pillars and trenches. The images were cropped to the regions above the pillars and these cropped images were used for segmenting cells using CellProfiler. For analysis, only the cells with centers within the ROIs were considered. Cellular orientation for cells in the ROIs was plotted as beeswarm plots for each mechanical stimulation condition (replicates pooled together but depicted in separate colors). In the beeswarm plots, the orientation of individual cells are plotted as a scatter with added spread where points overlap, making the plots appear thicker for angles along which a large number of cells are oriented. The beeswarm plots display the distributions of cell orientation angles with respect to the trench major axis. Thus, preferential alignment perpendicular to the maximum strain direction results in a bulge in the plot at 90° while preferential alignment along the flow produces a bulge in the plot at the angle that the trench major axis makes with the flow. Median values are marked with a solid blue line and a central ‘o’. Dotted red lines plus ‘|’ in the left corner and dotted green lines plus ‘*’ in the left corner mark the expected medians if the alignment was perfectly along the flow or perpendicular to the maximum principal strain respectively. Medians were calculated for orientation angles with respect to the reference line that would put the perfect alignment case at 90°, and were shifted appropriately if the reference line was not along the trench major axis.

In experiments conducted with HUVECs cultured on the device under media containing 5% wt/wt dextran, it was found that the cells aligned perpendicular to the maximum principal strain direction when strain alone was applied and along the flow direction when flow alone was applied. No alignment occurred in response to equibiaxial strains or in the static control, and the alignment in response to strain alone was significantly noticeable only for the higher degree of anisotropy strains. Interestingly, when flow and strain were simultaneously applied, cells aligned along the flow direction. Figure 5 shows the cell morphology for one replicate of each of the 25 conditions tested for the strain only and the flow and strain devices. Figure 6 shows the cell orientation angles for all conditions as beeswarm plots, where overlapping points in the distribution are spread apart. Thus the plots, which are a scatter of each individual cell’s orientation angle, are thicker (have a big spread) for the angles along which a large number of cells are aligned. For each condition, the replicates are pooled together but depicted in separate colors. The cell orientation angle distributions were multimodal, so medians were used to determine the general cell orientation direction. Figure 6 shows all cell orientation angles with respect to the respective trench major axis. The medians are marked with solid blue lines and a central ‘o’, dotted red lines and a left ‘|’ mark the orientation direction in the case of perfect alignment along the flow and dotted green lines along with a left ‘*’ mark the orientation direction in the case of perfect alignment perpendicular to the maximum principal strain direction. The blue lines were generally close to a red or a green line. Some cases where this did not happen were (i) in low density plots, i.e. conditions where few cells were present, (ii) in cases where there was no significant alignment (no prominent bulge in the distribution observed) and (iii) in cases where two lines were almost 180° apart, which was not a real difference since 0° and 180° angles represent the same alignment direction. A fourth case of a big difference between the red/green and the blue lines was observed for the flow and strain stimulation conditions with high degree of anisotropy strains aligned at intermediate angles (30°, 45° or 60°) to the flow. This indicates a mixed response of the cells to the two stimuli, where the alignment due to one signal is dominant (fluid flow shear stress in the case of high viscosity medium and strain anisotropy in the case of low viscosity medium), but a clear contribution of the other signal is present. At more extreme angles, this turned to a complete dominance of one stimulus when they opposed the other’s alignment response.

In experiments using media without dextran and a lower flow rate, cells aligned perpendicular to the maximum principal strain direction in response to simultaneous flow and strain stimulation. Flow only and strain only stimulation experiments performed with the low viscosity media had a shorter stimulation time (17 h, for the other experiments it was 24 h). However it was observed that the cells displayed feeble alignment along flow for the flow only stimulation and alignment perpendicular to the maximum principal strain for the strain only stimulation. No alignment was observed in the static control (Supplementary Figure 2).

## Discussion

Using a medium throughput device to apply various anisotropic strains at various orientations to flow induced shear stress, it was found that (1) when stimulated with strain alone, the alignment response of HUVECs is stronger for a higher degree of anisotropy, and (2) when combined strain and flow stimulation is applied, at a low shear stress of 0.08 Pa, alignment response is dominated by strain while at a higher shear stress of 0.55 Pa, alignment response is dominated by the shear stress. Enabled by the device developed here, these results demonstrate for the first time how endothelial cell alignment response is affected by anisotropic strain and shear stress combinations that do not stimulate alignment in the same direction. Since cell behavior and function often depends on the cell shape and orientation, this information can be important to better understand biological processes in complex mechanical environments.

The enhanced throughput ability of the reported device is essential for the testing of a large number of conditions in a few experiments. The ROIs on the device can be exposed to uniform anisotropic surface strains and shear stresses and have an area of 0.88 mm^2^ which allows for up to a few hundred attached cells to be studied. This is sufficient for statistical analysis and also makes the device suitable for co-culture experiments in the future. These features put the developed device in a unique position. Previously reported macroscale devices^21^ that can apply strains and flows simultaneously to cells are limited in throughput while in microscale^12^ devices the culture area and thus the number of cells per replicate is limited. Suspended membrane devices^26^, where strain actuation is provided by pulling attached walls perpendicular to the membrane, are restricted in the orientations between strain anisotropy and flow that can be tested due to the wall coming in the way. Shear stresses applied using cone and plate mechanisms^27^ or flow in tubes^22^,^24^ can be challenging to apply at varying orientations to strain. And out of plane membrane stretching to produce anisotropic strains is not straightforward to integrate with flow^28^.

Several aspects of the current device can be improved, including flow disturbances due to the strain actuating membrane dips, media pooling between dissimilar conditions, disturbed shear stresses near channel bends, limits on shear stress magnitude, and the relatively large amounts of reagents required compared to microscale devices. We demonstrated previously by comparing 0.5 mm and 2 mm high channels that a higher channel can reduce the variations in shear stresses due to the membrane dips during strain application^15^. Hence 2 mm high channels were used. However, still the deformed channel geometry models predicted shear stress variations of up to 15.7% from the undeformed state, making the flow effectively pulsatile in the ROIs. Since the variations could be quantified, an effectively pulsatile flow was not considered a major drawback. Apart from that, pulsatility can be theoretically diminished by varying the inflow over time to compensate for the volume increase induced by the moving membrane during the deformation cycle. The flow velocity change due to the extra volume generated by the membrane dips was minimized by keeping the overall flow rate high. Non-negligible flow velocity differences could still persist along the channel and hence replicates were kept in regions near inlet and near outlet (Fig. 1(c)) to detect any significant differences. Similarly, the near bends and mid-channel regions (Fig. 1(c)) housed replicates that could check for significant differences introduced by the shear stress disturbances near the bends. Pooling of the culture medium was tolerated assuming that in the primary mode of operation, i.e. combining strain and flow, the paracrine signaling molecules would be continuously washed off and highly diluted by the flowing medium. The need for large amounts of reagents such as cell culture medium cannot be avoided, but for reagents such as the staining solutions, the solutions could be reused for multiple devices.

Overall, the developed device provides unprecedented possibilities to study the underexplored effects of anisotropic strain and flow combinations on cells. The device is easy to use and its flexible design allows for future innovations in overcoming existing limitations and addition of functionality such as surface patterning to selectively allow cell attachment in ROIs. The data generated with this device, as shown here for the alignment of HUVECs to combinations of anisotropic surface strains and fluid flow shear stresses, is likely to provide unique insight into the importance of combined mechanical signals for cell behavior and tissue development both *in vivo* and during *in vitro* cell culture.

## Methods

### Device fabrication

The device was fabricated by gluing together layers which were produced by casting PDMS (Sylgard 184, 10:1 base: curing agent) in molds and curing at room temperature for 48 h. Three PDMS layers were formed in this way – (i) a pillar array, (ii) fluid flow channels and (iii) a trench-air-channel layer. The molds for the pillar array and the fluid flow channels were made by laser cutting transparent poly-methylmethacrylate (PMMA) sheets (Snijlab). The trench-air-channel mold was prepared by milling aluminum.

The fabrication steps, as illustrated in Supplementary Figure 3, were as follows. A PDMS membrane was prepared by spin coating (5 s at 100 rpm followed by 40 s at 1000 rpm) a mixture of PDMS and 6 μm polystyrene beads (Polysciences Inc.) on a perfluorodecyltrichlorosilane (FDTS) coated silicon wafer (Siegert Wafer GmbH). The membrane was cured at 60 °C for 2 h and then the trench-air-channel layer was attached to it using uncured PDMS as glue, which was spin coated on a silicon wafer (120 s at 1500 rpm) and stamped on the non-air-channel side of the trench-air-channel layer. The pillar array was plasma bonded to a 1 mm thick glass base plate and all pillar tops were covered with drops of silicone oil lubricant (Griffon HR260 silicone spray). The trench-air-channel with the membrane was taken off of the silicon wafer, aligned with the lubricated pillar array, and attached to it without a glue. The device parts were stored at this point. Just before the cell studies, the top of the membrane and the inside of the flow channels were plasma treated and glued together using NOA81 (UV curable glue, Norland Products Inc.). The plasma treatment and the UV curing of the NOA81 served an extra purpose of sterilization along with making the surface hydrophilic and bonding the layers. The tubing was attached inside a sterile laminar flow cabinet and the device was clamped between a laser cut steel plate on top and a 2 mm thick glass plate at the bottom to avoid leaks during operation. The top steel plate contained openings above the channels to allow for gas exchange through the PDMS (Supplementary Figure 3(e)). Once the clamped device was ready, it was filled with a fibronectin solution (0.5 μg/ml) in phosphate buffered saline (PBS) and incubated at room temperature for at least 1 h. Fluid was filled in the device using a syringe and with a small tilt to the device to keep the fluid front moving against gravity, such that any bubbles moved to the fluid front and were finally pushed out of the outlet. The fibronectin solution was removed just before filling the device with a cell suspension.

The important dimensions of the developed prototype are as follows: (i) base 127.8 mm × 85.5 mm (same as standard well plate base dimensions), (ii) pillar and trench height 1 mm, (iii) air channel height 0.5 mm, (iv) fluid flow channel height 2 mm, (v) pillar radius 0.75 mm, (vi) membrane thickness 80 μm, (vii) circular trench radii 1.25, 1.5, 1.75, 2 and 2.5 mm and (viii) ellipse trench dimensions (half of minor axis length – half of major axis length) 1.55–2.3, 1.7–2.3, 1.9–2.3 and 2.1–2.3 mm.

### Computational modeling of strains and fluid flow

Computational models of the strain unit and the flow channels were analyzed using ANSYS 13.0 Workbench (ANSYS Inc). For the strain, a quarter unit was modeled with symmetry boundary conditions. To analyze the overall shear stress distribution, the complete flow channel network was modeled. To analyze the local changes in shear stress due to the strain actuating deformation of the membrane, flow was modeled in deformed channel geometries for the various unit types at various applied pressure drops.

The strain models consisted of three parts – a circular pillar (1 mm high, 0.75 mm radius), a membrane (80 μm thickness) and a part of the trench-air-channel layer with various trench dimensions. All parts were modeled as PDMS with isotropic material properties. The elastic modulus used was 1.84 MPa and the poisson’s ratio used was 0.499^29^. All contacts were modelled as bonded contacts except the pillar-membrane contact, which was modelled as a frictional contact with a coefficient of friction of 0.01^15^. Fixed contact boundary condition was applied to the base of the pillar and the trench-air-channel layer and a pressure drop was applied to the bottom of the membrane. A linear pressure profile going from 0 to 20 kPa over 1 s was used. The principal strain vectors were exported and averaged for the ROIs.

For the flow models, a velocity inlet, a pressure outlet and a no-slip wall were used as boundary conditions. The fluid properties used were – 1000 kg/m^3^ density and a 0.001 Pa.s (cell culture media) or a 0.0037 Pa.s viscosity (viscosity of cell culture media with 5% wt/wt dextran). The viscosity for the media alone was taken from literature^30^ and the viscosity of dextran containing media was determined by comparing the time taken by it to flow across a tubing with a fixed applied pressure drop with the time taken by media without dextran. Inlet velocities used were 0.1 m/s or 0.2 m/s for the full channel (corresponding with 20 ml/min or 40 ml/min respectively) and 0.04 m/s for the deformed geometries (corresponding with 40 ml/min). A 0 Pa pressure was applied to the outlet in all cases. Once the models were solved, wall shear stress values were exported and averaged over the ROIs.

The deformed channel geometries were created using the strain modeling results. The maximum dip of the membrane for the circular trench or the maximum dips along the trench major and minor axes were calculated and used to create the deformed geometries using SolidWorks (Dassault Systemes). For the circular trenches, the deformed membrane geometry was created by approximating the membrane deformation as a circular arc with the calculated dip. For the ellipse trenches, two circular arcs with the calculated dips were drawn along the trench axes and a loft feature passing through them was created.

### Device characterization

Strains were empirically determined by tracking the beads embedded in the membrane. Strains were applied using an Elveflow AF1 dual pressure-vacuum generator and controller (Elvesys Innovation Center, France). Images of the membrane over the pillars were taken at 0, 5, 10 and 20 kPa pressure drops. Bead co-ordinates in the images were determined by using a local maxima/minima detection following a Gaussian blur (2 pixel radius) in ImageJ (NIH). The detected beads were mapped between images using a custom Matlab (MathWorks Inc.) script to assign the same number to beads in all images^15^. Strains were determined for all pairs of beads as the ratio of the change in distance between a bead pair to the initial distance between the bead pair. The bead pair strains were plotted against the angle that the line joining the bead pair made with the trench major axis (bead pair orientation angle). Strains were averaged for bead pairs with orientation angles within 2.5° on either side of the major axis and the minor axis and these averaged strains were used as approximations for the maximum and the minimum principal strains respectively.

### Cell studies

The tubing setup during cell studies was similar as reported previously^15^. Primary HUVECs under passage 10 were used for the cell studies. The cells were expanded in T75 flasks using endothelial growth media from a bullet kit (EGM2, Lonza). Cells were seeded at a high density (>20,000 cells/cm^2^) to get a confluent cell layer on attachment. After filling the device with the cell suspension, the device along with the tubing was moved to a 37 °C, 5% CO_2_ incubator and left there for 18–20 h allowing the cells to attach. The cells were then stained with CellTracker Green CMFDA (ThermoFisher Scientific) using the manufacturer’s protocol and imaged in the ROIs. Mechanical stimulation was then applied for 24 h total, with one break after ~6 h of stimulation, to check the cells. Separate devices were used to apply flow only, strain only, flow and strain, or no stimuli (static control) to cells. Strains were applied using an Elveflow AF1 system – a sinusoidal pressure profile going between 0 and 20 kPa with a 1 s period was used. Flow was applied using a Legato 270P syringe pump (KD Scientific) – a 20 ml/min or a 40 ml/min volumetric flow rate was applied. The syringe pump used two 50 ml syringes (BD Plastic), one pushing fluid into the device while the other filled, and one-way check valves ensuring that fluid was filled from the reservoir and pushed into the device by both syringes. Thus a continuous flow was maintained, except for the brief interval (0.25 s according to pump manufacturer) when the pump switched directions. This occurred at 1 minute intervals for the 20 ml/min flow and at 30 s intervals for the 40 ml/min flow. The syringes were replaced after every 12 h of use in order to avoid piston failure due to friction build up. After 24 h of stimulation, the cells were fixed with 4% paraformaldehyde. They were then stored at 4 °C under PBS until the cells could be stained. For staining the cells, they were first washed with PBS, followed by 1% Triton X-100, then again by PBS and then blocked for 1 h with a 5% bovine serum albumin (BSA) solution in PBS to prevent non-specific staining. Staining for filamentous actin was done using Alexa 488 Phaloidin (solution in 5% BSA) for 1 h followed by staining for DNA using DAPI (solution in 5% BSA) for 20 minutes. The cells were then washed with PBS and water and stored dry until imaging. Automated imaging was performed for all ROIs using BD Pathway (BD Biosciences).

### Image analysis for cell alignment quantification

Images captured were 3 × 3 montages acquired using a 4× objective. The montages covered the entire pillar and trench area around a ROI. Two montages were taken per ROI, one each with the fluorescence excitation and emission filter settings for Alexa 488 (cell image) and DAPI (nuclei image). A custom Matlab script was used to crop images to the regions above the pillars by detecting the pillar edge in the nuclei images. The pillar edge was always darker than the rest of the image, allowing the Matlab script to search for the center of a circle with the same radius as the pillar edge, that had the lowest cumulative intensity along its circumference. An image mask was then applied to black out all area outside the pillar area. Background subtraction was performed to remove effects of non-uniform illumination and contrast enhancement using ImageJ. CellProfiler^31^ was then used to segment the nuclei and the cells and measure their shape and size. The CellProfiler pipeline first used thresholded nuclei images to identify the nuclei as primary objects and then the cells were segmented in the cell images as secondary objects for each nucleus using the propagation algorithm. The shape and size of the segmented cells were exported from CellProfiler and analyzed for the cells whose center was within the ROI. Artifacts falsely identified as cells were removed using lower and upper size filters combined with a filter for eccentricity.

Cell orientation angles were plotted as beeswarm plots and the median values were marked. For determining the medians, cell orientation angles with respect to the image horizontal direction (perpendicular to flow) were used when cells aligned to flow and with respect to the trench major axis when cells aligned to strain. This ensured that the bulge in the beeswarm plots occurred near the middle of the distribution and the medians represented the majority alignment direction.

## Additional Information

**How to cite this article**: Sinha, R. *et al.* Endothelial cell alignment as a result of anisotropic strain and flow induced shear stress combinations. *Sci. Rep.* **6**, 29510; doi: 10.1038/srep29510 (2016).

## Supplementary Material

Supplementary Information

## Figures and Tables

**Table 1:** The developed device can apply 25 separate mechanical stimulation conditions to cells.

Condition number	Strain condition number	Trench shape	Trench dimensions	Orientation angle of trench major axis with respect to flow
1	1	circular	radius 1.25 mm	–
2	2	circular	radius 1.5 mm	–
3	3	circular	radius 1.75 mm	–
4	4	circular	radius 2 mm	–
5	5	circular	radius 2.5 mm	–
6	6	ellipse	half minor axis 1.55 mm half major axis 2.3 mm	0°
7				30°
8				45°
9				60°
10				90°
11	7	ellipse	half minor axis 1.7 mm half major axis 2.3 mm	0°
12				30°
13				45°
14				60°
15				90°
16	8	ellipse	half minor axis 1.9 mm half major axis 2.3 mm	0°
17				30°
18				45°
19				60°
20				90°
21	9	ellipse	half minor axis 2.1 mm half major axis 2.3 mm	0°
22				30°
23				45°
24				60°
25				90°

The conditions are listed here and assigned a condition number each for ease of identification. In the absence of flow, the variously oriented strain conditions are identical and hence a strain condition number is also defined to collectively refer to these identical strain conditions.
