# Supplementary material for: Endothelial cell alignment as a result of anisotropic strain and flow induced shear stress combinations
Source: Sci Rep. 2016 Jul 12;6:29510. doi: 10.1038/srep29510 (PMC4941569; doi:10.1038/srep29510)
Supplement: Supplementary Information [file srep29510-s1.pdf]

Endothelial cell alignment as a result of anisotropic strain and flow induced shear stress combinations

Ravi Sinha[1], Séverine Le Gac[2], Nico Verdonschot[1,3], Albert van den Berg[4], Bart Koopman[1] and Jeroen Rouwkema[1]*

[1] Department of Biomechanical Engineering, MIRA Institute for Biomedical Technology and Technical Medicine, University of Twente, Enschede, The Netherlands

[2] Applied Microfluidics for BioEngineering Research group, MIRA Institute for Biomedical Technology and Technical Medicine, MESA+ Institute for Nanotechnology, University of Twente, Enschede, The Netherlands

[3] Radboud university medical center, Radboud Institute for Health Sciences, Orthopaedic Research Lab, P.O. Box 9101, 6500 HB, Nijmegen, The Netherlands

[4] BIOS, Lab on a chip group, MIRA Institute for Biomedical Technology and Technical Medicine, MESA+ Institute for Nanotechnology, University of Twente, Enschede, The Netherlands

E-mail: j.rouwkema@utwente.nl

Supplementary Information

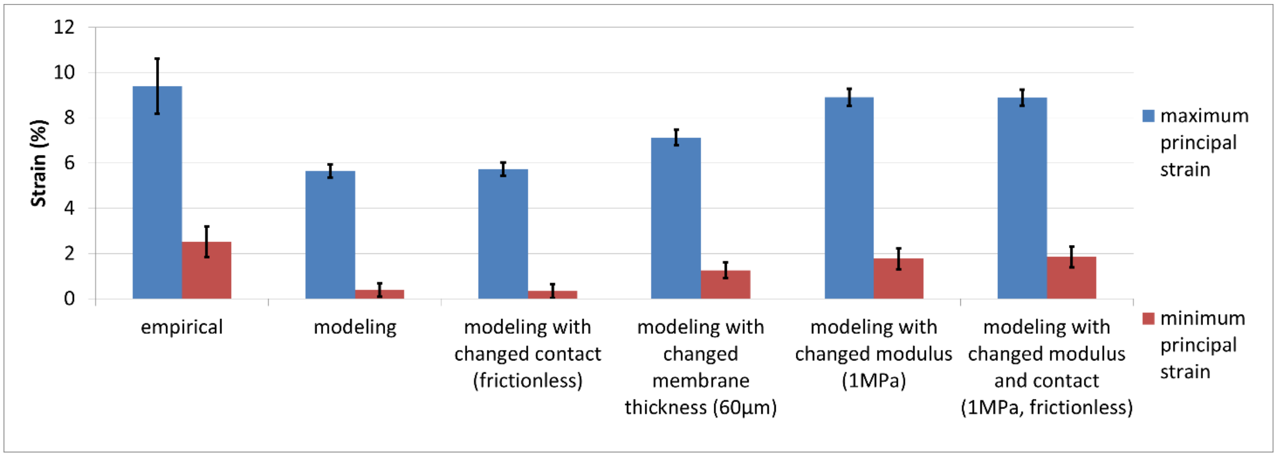

Supplementary figure 1 – Strain (at 10 kPa) value comparison between empirical and modeling results for the strain condition number 6. Changing the friction between the membrane and the pillar or varying the membrane thickness modified the strain values by small amounts, but changing the modulus of PDMS brought the modeling strain values very close to the empirical value. Averages ± standard deviations are shown for all strain values. For the empirical results, the averages and standard deviations are calculated for 20 replicates, while for the models, they depict the local variation within a single ROI.

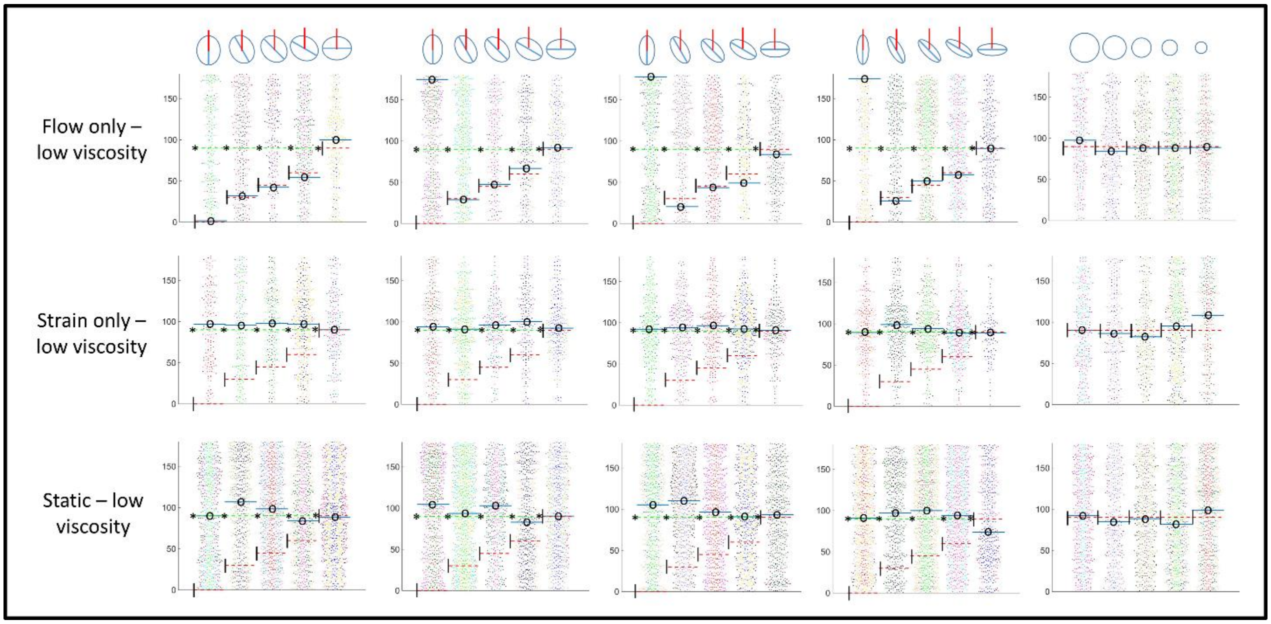

Supplementary figure 2 – Beeswarm plots of the cell orientation angles are shown for the low viscosity medium. Solid blue lines and a central 'o' mark the median values, dotted red lines and a left '|' mark the flow direction and dotted green lines with a left '' mark the direction perpendicular to the maximum principal strain.*

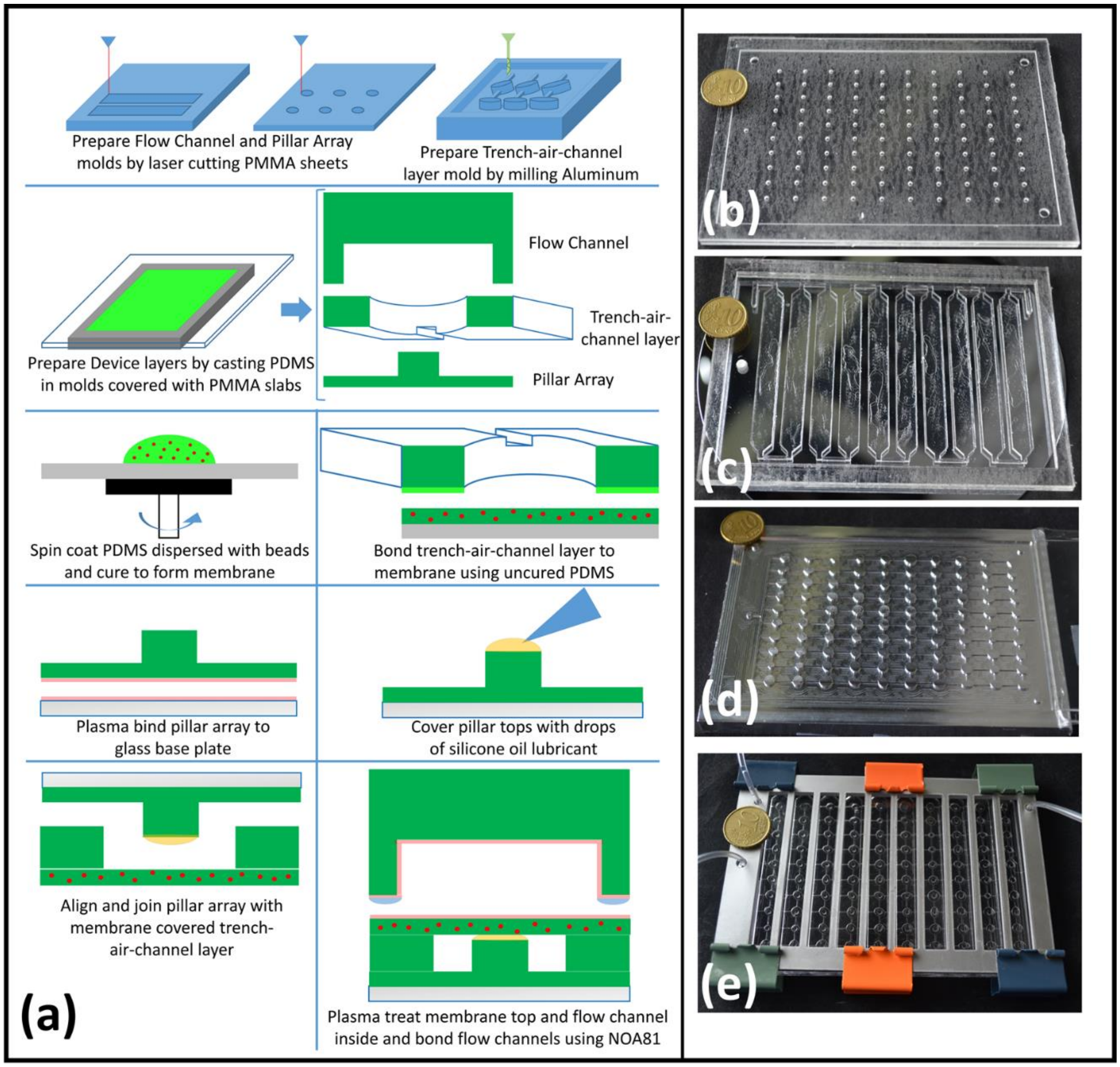

Supplementary figure 3 – The fabrication process is presented schematically (a). The device layers were made from three molds – pillar array (b), flow channel (c) and trench-air-channel (d) molds. After channels were attached, the device was clamped before filling and using (e).
